# Effect of Potassium Permanganate as an Ethylene Scavenger and Physicochemical Characterization during the Shelf Life of Fresh Banana (*Musa paradisiaca*)

**DOI:** 10.1155/2023/4650023

**Published:** 2023-08-22

**Authors:** Brayan Ronaldo Gutierrez-Aguirre, Ramiro Enmanuel Llave-Davila, Luis Alberto Olivera-Montenegro, Esteban Herrera-Nuñez, Luis Alejandro Marzano-Barreda

**Affiliations:** Grupo de Ciencia, Tecnología e Innovación en Alimentos, Universidad San Ignacio de Loyola, Lima 15024, Peru

## Abstract

The conventional method of employing low temperatures for storage and distribution has long been the standard approach for preserving most fruits and vegetables. This practice is likewise prevalent in the retail industry, which relies on similar methods for transporting and maintaining the quality of perishable products on their shelves. The aim was to preserve bananas (*Musa paradisiaca*) using an ethylene scavenger, potassium permanganate, which is contained in small paper bags, to increase the storage and distribution time at low cost. The bananas were distributed in four plastic containers at a temperature of 23°C, three of the treatments contained different concentrations of potassium permanganate, and one was potassium permanganate free. The experimental period was 19 days, and the variations in weight loss, pH, titratable acidity, texture, color, and total soluble solids were analyzed. Potassium permanganate effectively reduced the changes in their physiological ripening.

## 1. Introduction

Historically, the Musa species was developed due to the migration of populations that range from New Guinea to West Africa, which favored multiple changes in its genotype. This generated the natural hybrids that we know today [1].

The banana of the Musaceae family has been one of the most important commercial crops in tropical countries. The nutritional value is one of the main characteristics of this fruit. Banana-producing countries have increased their banana-growing areas along with the banana-processing industry [2].

The postharvest management of banana is mainly conditioned by the control of the level of ethylene released, using compounds such as ethephon which induce the increase of ethylene production present in the fruit, promoting physicochemical changes of ripening [3]. Maintaining low ethylene levels is also a strategy that serves to decrease product loss and maintain product quality [4].

The fruit-ripening process produces changes in quality attributes related to ethylene production, such as flavor, texture, aroma, and nutritional values. In turn, this process results in multiple molecular events directly related to the manifestation of ethylene [5].

Food product losses are a worldwide problem every year. According to FAO [6], approximately 1.3 billion tons per year of food produced for human consumption was wasted. The retail industry has been mainly responsible for wastes such as fruits, vegetables, and meats that are discarded daily in the process of shelf sanitation.

Preservation methods emerged as prevention of product losses using techniques such as low temperatures, atmosphere modifiers, and growth regulators such as 1-methylcyclopropene (C_4_H_6_) which has been reported as an effective method to prolong the shelf life of the product and delay ripening of a wide variety of climacteric fruits [7, 8].

The application of ethylene scavengers represents an effective preservation strategy for climacteric fruits; we can highlight the use of potassium permanganate (KMnO_4_). These scavengers were developed to reduce the cost of traditional preservation maintenance such as refrigeration, whose value is higher due to the parameters in which they must operate [9].

Potassium permanganate (KMnO_4_) physically absorbs the surrounding ethylene through a porous medium, oxidizing it and forming manganese oxide, potassium hydroxide, water, and CO_2_. During the process, the color of the KMnO_4_ changes from purple to brown which indicates that the absorption capacity is complete [10].

The disadvantages of using KMnO_4_ as an ethylene scavenger are that it can remove an aroma that is desired to be present in the fruit, in addition to the fact that it is possible to migrate KMnO_4_ directly into the product; it should be noted that this contains a high degree of toxicity even when it is purple [11].

Treatments with KMnO_4_ in bananas have been effective in slowing down senescence and maturity, increasing their shelf life, and maintaining fruit quality when stored. Banana firmness is the main attribute maintained when treated with a specific dose of potassium permanganate in a warehouse-type environment [12].

Other types of ethylene scavengers are zeolite, silica, and activated carbon. They are usually found in small bag presentations that are constantly replaced due to saturation to keep it in a controlled environment [4].

Studies using KMnO_4_ successfully maintained good appearance, firmness, and reduced weight loss in peach [13], delayed degreening and maintained the firmness in apple [14], and reduced weight loss and delayed ascorbic acid degradation in tomatoes [15]. For the reasons explained above, this study was aimed at evaluating the effect of potassium permanganate on bananas stored at 23°C.

## 2. Materials and Methods

### 2.1. Materials

Potassium permanganate in small wax-coated paper bags (5 g) (Bion®, Spain) was provided for the study.

### 2.2. Experimental Procedure

The experiment was a completely randomized design and was carried out in a total of 19 days where, on days 1, 3, 7, 12, and 19, the following parameters were measured: titratable acidity, color, weight loss, pH, texture, and total soluble solids. The study was carried out with 4 plastic containers (Rey Plast®, Peru), each containing 7 kg of banana. The bananas (*Musa paradisiaca* Cavendish cv. Seda) were harvested in the Junin region located at 3500 meters above sea level with coordinates 11°17′S latitude, 74°31′E, near the Mazamari district, and purchased in the fruit market of the San Luis district (Lima, Peru) at a low ripening point (green color). Four treatments were processed including a control treatment: 0.07%, 0.14%, and 0.21% of potassium permanganate and bananas without potassium permanganate. These percentages were recommendations from the provider (Bion®, Spain) and are related to the weight of the fruit. Each experimental unit contained 3 bananas; this is to reduce the area of action of the potassium permanganate and develop a controlled storage environment. The experiment was carried out in duplicate.

### 2.3. Physiological Weight Loss

Weight loss was calculated using the following formula.

(1) $$\begin{array}{c} W_{0}\left(\%\right)=\frac{W_{0}-W_{t}}{W_{0}}\times 100, \end{array}$$

where *W*_0_ (%) is the percentage weight loss, *W*0 is the initial weight of the fruit, and *W*_*t*_ is the weight of the fruits at the designated time.

### 2.4. Fruit Firmness

The peel of bananas was removed, and the sample was prepared by cutting the pulp transversely dividing it into 1 cm wide and 2.5 cm high. The banana firmness was determined using a TA.HD plus–TA.XT texturometer (Stable Micro Systems, England). The required force was measured using a 0.2 cm cylinder probe (P/3) used to penetrate to a distance of 0.4 cm at the rate of 0.1 cm/s. The load was 5 kg. The results were expressed as N.

### 2.5. Peel Color Change

The peel color was measured with the colorimeter (PCE-CSM 10, Germany), which was regularly calibrated before measurement with a standard black-and-white panel and illuminant D65. The color change of the sample fruit was determined using the CIE (Commission Internationale de L'Eclairage) 1976 L^∗^a^∗^b^∗^ color scale system, where the L^∗^ scale represents lightness (“dark vs. light”) where one hundred is for full white and zero for full black, a^∗^ measures the variation of red (“red vs. green”), b^∗^ measures blue to yellowish, and h° represents hue. 21 measurements were captured for each finger in the unit treatment for the 4 treatments and their duplicates.

### 2.6. Total Soluble Solids (TSS) and Titratable Acidity (TA)

The titratable acidity (TA) of the pulp was measured using the formula adopted by Al-Dairi et al. [16] . For the study, 50 g of pulp, taken from the cross-section of the fruit, was placed in 150 mL of distilled water, blended for 2 min in a kitchen blender (BLSTBESTE-053, Oster®, USA), and then filtered through filter paper (Whatman®, USA). A drop of the filtrate was placed on the prism of a digital refractometer (Hanna®, HI 96812, USA), and the amount in °Bx was noted. Ten mL of the filtrate was extracted and mixed with 90 mL of distilled water; then, 5 drops of the phenolphthalein indicator (Similaxol, Mexico) were added. Then, it was titrated with NaOH 0.1 N (Merck Millipore, USA). The volume of NaOH used was noted until the indicator turned pink.

(2) $$\begin{array}{c} \text{Titratable acidity }\left(\%\right)=\frac{V_{1}*N*E}{V_{2}}\times 100, \end{array}$$

where *N* is the normality of NaOH, *V*_1_ is the volume of NaOH used, *E* is the equivalent weight of acid, and *V*_2_ is the volume of the sample taken for estimation.

### 2.7. pH

The acidity verification was performed using a digital pH meter (HANNA®, HI 8424, USA) that was previously calibrated at the beginning of the day. The 50 g sample of the banana was liquefied and then directly analyzed.

### 2.8. Statistical Data Analysis

Data were analyzed using Statistica 8.0 (StatSoft, USA), with analysis of variance (ANOVA) and Tukey's test at 5% confidence.

## 3. Results and Discussion

### 3.1. Physiological Weight Loss

**Figure 1:**
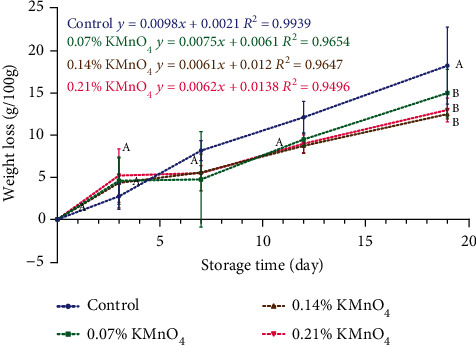
Weight loss of banana during 19 days (control, 0.07% KMnO_4_, 0.14% KMnO_4_, and 0.21% KMnO_4_). ^A,B^Different letters on the same day indicate significant differences (Tukey test *p* ≤ 0.05).

The percentage change of weight loss was described by a zero-order kinetic model in Figure 1. The control response factor (*R*^2^) was calculated to be 0.99. The closer the value was to it, the more accurate the control. Treatment reduces this response factor in relation to the concentration of KMnO_4_.

The weight-loss rates were 0.0098%, 0.0075%, 0.0061%, and 0.0062% per day for bananas from the control treatment, 0.07% KMnO4, 0.14% KMnO4, and 0.21% KMnO4, respectively. According to Li et al. [17], weight reduction is identified as a potential factor influencing the ripening process, which is regulated by the hormone ethylene. The presence of the scavenger KMnO_4_ has been demonstrated by Deshmukh et al. [18] to decrease ethylene levels. Consequently, the variations in ripening rates may be attributed to the varying concentrations of KMnO_4_.

The control treatment had the highest rate of weight loss of 0.0098%/day, while the KMnO_4_ treatments reduced that loss by up to 31%; this is consistent with the banana study presented by Deshmukh et al. (see [17]) who report that the effect of KMnO_4_ helped reduce weight loss from 4.48% to 2.96% in the first 8 days postharvest in bananas.

### 3.2. Fruit Firmness

Table 1 shows the reduction in the firmness during the 19 days of the experimental study. The most noticeable difference occurred on day 12 where all the treatments with KMnO_4_ achieved better results than the control treatment. Firmness is one of the principal quality parameters that can determine the acceptance of the product by the target public. Alonso-Salinas et al. [19] showed that different changes in banana firmness depend on the combination of conservation technologies.

Based on a similar study, Bal and Celik [20] reported that the “Hayward” kiwi maturity process can be delayed by using potassium permanganate (KMnO_4_). Also, Álvarez-Hernández et al. [21] affirm that KMnO_4_ scavengers are effective tools to delay the pulp firmness loss in apple fruit, as well as to reduce sugars, free organic acid, and physiological disorder content throughout storage at low temperatures.

### 3.3. Peel Color Change

**Figure 2:**
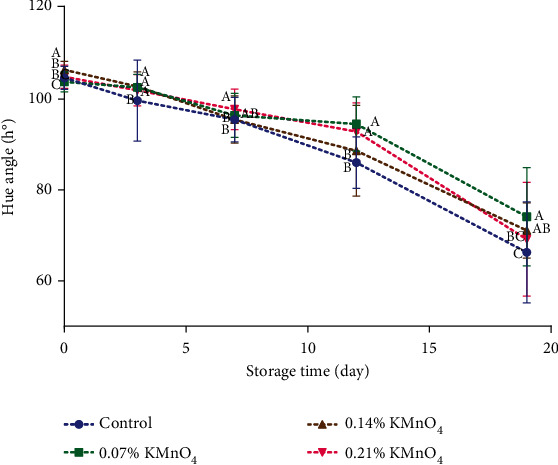
Color (hue value) of banana during 19 days (control, 0.07% KMnO_4_, 0.14% KMnO_4_, and 0.21% KMnO_4_). ^A,B,C^Different letters on the same day indicate significant differences (Tukey test *p* ≤ 0.05).

**Figure 3:**
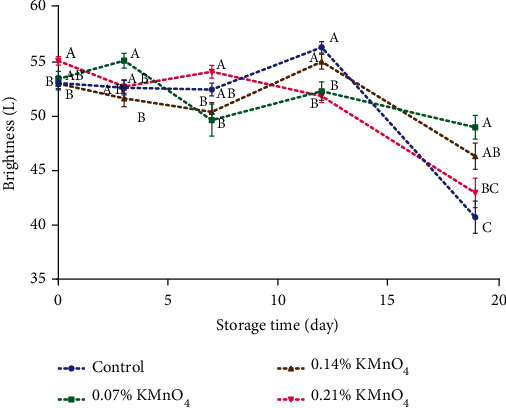
Color (lightness value) of banana during 19 days (control, 0.07% KMnO_4_, 0.14% KMnO_4_, and 0.21% KMnO_4_). ^A,B,C^Different letters on the same day indicate significant differences (Tukey test *p* ≤ 0.05).

In the results of the change of the hue angle (h°) and brightness (L^∗^) (Figures 2 and 3), it was possible to observe significant differences (*p* ≤ 0.05) between the treatments.

During the storage period, there was a reduction in the values of both hue angle and brightness. Specifically, on the nineteenth day, the control treatment showed the most significant decrease in both parameters, followed by the treatment with 0.14% KMnO_4_, then the treatment with 0.21% KMnO_4_, and finally the treatment with 0.07% KMnO_4_.

For the banana under natural ripening condition, ethylene gas is autostimulated by the ripening fruit. In this case, the fruit ripens slowly and unevenly causing high weight loss and desiccation and failing to develop good color and aroma [22]. Mejia [23] confirmed that the change in the peel color (hue angle and brightness) from green to yellow during the ripening stage is due to the decrease of chlorophyll by enzymatic activity hydrolyzing from chlorophyll and phytol. Also, Ademe and Tanga [24] reported that bananas wrapped in moringa leaves resulted in higher starch and chlorophyll hydrolysis probably leading to faster coloration of the peel.

The color change of the banana peel depends on internal and external factors. In this study, potassium permanganate at low concentrations helped to decrease the effect of color change; however, at high concentrations, it has an inverse effect on banana peels [25].

### 3.4. Total Soluble Solids (TSS)

A significant variation was observed in this parameter (*p* ≤ 0.05) among the treatments throughout the storage time (Table 2). A significant variation was observed in this parameter (*p* ≤ 0.05) between the treatments and days (Table 3).

All the results for soluble solids follow the same pattern, with control treatment being the one with the highest °Bx on all days, followed by 0.14% KMnO_4_ and 0.21% KMnO_4_ and finally 0.07% KMnO_4_. This increase is due to the hydrolysis of starch into sugar [26]. The difference between treatments indicates that KMnO_4_ contributed to delay respiration and the conversion of starch to sugar.

### 3.5. Titratable Acidity (TA)

A significant variation was observed in this parameter (*p* ≤ 0.05) among the treatments throughout the storage time (Table 2).

The observed results reveal a statistically significant distinction in the day-to-concentration ratio, whereas no statistically significant distinction is detected in the concentration variable alone. This suggests that employing the lowest concentration level in the experiment leads to a satisfactory outcome. The findings provide valuable insights for experimental design and emphasize the significance of carefully considering the day-to-concentration ratio for optimal experimental outcomes (Table 3).

Botrel et al. [27] reported that the increased value of the titratable acidity was related to the predominance of malic acid contained in the banana. The highest values were obtained when the peel of the banana turned yellow, and then, these values decrease until senescence.

### 3.6. pH

A significant variation was observed in this parameter (*p* ≤ 0.05) among the treatments throughout the storage time (Table 2). In all treatments, the pH level decreased progressively over time.

The results obtained agree with Fatemeh et al. [14] who reported that the use of KMnO4 on golden apples keeps pH values low.

The results agree with those reported by Tourkey et al. [28]: as the storage period progresses, total acidity increases resulting in a decrease in the pH of the fruit. Similar results were reported by Wang et al. [29] who showed that the pH values decrease after harvest.

## 4. Conclusions

The findings from the two-way analysis of variance (ANOVA) provide substantial evidence supporting the existence of a significant distinction between the various days and treatments (0.07%, 0.14%, and 0.21%), with the exception of the %A.C. analysis, where the dissimilarity is negligible or nonexistent.

The evidence provided by these results strongly suggests that the 0.07% treatment showed an advantage over the alternative treatments, displaying a capability in preserving color, total soluble solids, and firmness.

This study needs to be scaled up for storage in retail industry warehouses without resorting to storage methods such as refrigeration to increase product shelf life at a lower cost.

## Figures and Tables

**Table 1:** Firmness of banana during 19 days (control, 0.07% KMnO_4_, 0.14% KMnO_4_, and 0.21% KMnO_4_).

Treatments		Day 3	Day 7	Day 12	Day 19
0.21% KMnO_4_	N	8.31 ± 0.374^b^	8.49 ± 0.815^a^	6.90 ± 1.536^b^	0.61 ± 0.159^b^
0.14% KMnO_4_	N	8.31 ± 0.986^b^	7.92 ± 0.876^b^	6.08 ± 3.231^c^	0.73 ± 0.247^a^
0.07% KMnO_4_	N	8.68 ± 0.358^a^	7.39 ± 1.730^c^	7.93 ± 1.070^a^	0.63 ± 0.172^b^
Control	N	7.56 ± 0.660^c^	7.82 ± 0.639^b^	3.62 ± 2.912^d^	0.45 ± 0.193^c^

^a,b,c,d^Different letters on the same day indicate significant differences (Tukey test *p* ≤ 0.05).

**Table 2:** Acidity (pH), total soluble solids (°Bx), titratable acidity (%A.C.) of banana during 19 days (control, 0.07% KMnO_4_, 0.14% KMnO_4_, and 0.21% KMnO_4_).

Treatments		Day 0	Day 3	Day 7	Day 12	Day 19
0.21% KMnO_4_	pH	5.61 ± 0.081^a/vw^	5.60 ± 0.130^ab/v^	5.46 ± 0.080^a/wx^	5.39 ± 0.150^a/x^	4.99 ± 0.100^b/z^
	°Bx	3.50 ± 0.157^a/w^	2.86 ± 0.083^c/w^	3.73 ± 0.065^b/w^	4.70 ± 0.422^b/w^	18.33 ± 0.840^a/v^
	%A.C.	0.48 ± 0.067^a/z^	0.48 ± 0.065^a/xz^	0.29 ± 0.063^a/w^	0.55 ± 0.157^ab/wx^	0.67 ± 0.083^ab/v^
0.14% KMnO_4_	pH	5.55 ± 0.080^a/v^	5.69 ± 0.070^a/v^	5.40 ± 0.040^a/w^	5.30 ± 0.240^a/w^	4.86 ± 0.090^b/x^
	°Bx	3.67 ± 0.142^a/w^	3.40 ± 0.100^ab/w^	4.33 ± 0.111^ab/w^	6.13 ± 1.012^ab/w^	17.03 ± 1.402^a/v^
	%A.C.	0.41 ± 0.067^a/x^	0.40 ± 0.070^b/x^	0.24 ± 0.052^a/w^	0.62 ± 0.065^a/vw^	0.72 ± 0.083^a/v^
0.07% KMnO_4_	pH	5.61 ± 0.070^a/v^	5.55 ± 0.082^b/vw^	5.36 ± 0.120^b/x^	5.46 ± 0.034^a/wx^	4.89 ± 0.170^b/z^
	°Bx	3.63 ± 0.138^a/w^	3.16 ± 0.131^bc/w^	4.46 ± 0.272^a/w^	3.76 ± 0.116^b/w^	16.26 ± 0.736^a/v^
	%A.C.	0.48 ± 0.067^a/xz^	0.44 ± 0.070^ab/z^	0.26 ± 0.055^a/wx^	0.54 ± 0.238^b/vw^	0.69 ± 0.112^b/v^
Control	pH	5.55 ± 0.090^a/v^	5.63 ± 0.082^ab/v^	5.47 ± 0.020^a/v^	5.36 ± 0.430^a/vw^	5.18 ± 0.250^a/w^
	°Bx	3.83 ± 0.243^a/x^	3.70 ± 0.075^a/x^	4.60 ± 0.131^a/x^	7.43 ± 0.511^a/w^	19.56 ± 0.281^a/v^
	%A.C.	0.41 ± 0.067^a/x^	0.42 ± 0.048^ab/x^	0.25 ± 0.045^a/w^	0.76 ± 0.070^a/v^	0.58 ± 0.065^b/w^

^a,b,c^Different letters on the same day indicate significant differences (Tukey test *p* ≤ 0.05); ^v,w,x,z^different letters in the same rows indicate significant differences (Tukey test *p* ≤ 0.05).

**Table 3:** Two-way analysis of variance (ANOVA) hypothesis of acidity (pH), total soluble solids (°Bx), and titratable acidity (%A.C.).

	°Bx	%A.C.	pH
Days	*h*_1_	*h*_1_	*h*_1_
Treatments	*h*_1_	*h*_0_	*h*_1_
Days/treatments	*h*_1_	*h*_1_	*h*_1_

*h*_1_: significant differences between the parameters; *h*_0_: no significant differences between the parameters.

## Data Availability

The numerical data used to support the findings of this study are available from the corresponding author upon request. Identified as Luis Alejandro Marzano-Barreda whose email contact is lmarzano@usil.edu.pe.

## Abbreviations

0.07% KMnO_4_: 5 g of KMnO_4_ in 7000 g of banana
0.14% KMnO_4_: 10 g of KMnO_4_ in 7000 g of banana
0.21% KMnO_4_: 15 g of KMnO_4_ in 7000 g of banana.

## Additional Points

*Research Highlights*. (i) The use of potassium permanganate proved to be an efficacious approach for ethylene scavenging without requiring direct contact with the fruit. (ii) It was not necessary to use low temperatures for the conservation of fruits and vegetables, as is currently used by the retail industry, reducing transportation and storage costs. (iii) Potassium permanganate was efficient in reducing the metabolism of fresh bananas stored at 23°C preserving the titratable acidity and color and having a low weight loss rate and low total soluble solids for 19 days.
